# Evaluating an Education Program to Reduce Indeterminate QuantiFERON Gold In-Tube Results

**DOI:** 10.1155/2018/7906846

**Published:** 2018-10-11

**Authors:** Saroochi Agarwal, Duc T. Nguyen, Justin D. Lew, Brenda Campbell, Edward A. Graviss

**Affiliations:** ^1^Houston Methodist Research Institute, Department of Pathology and Genomic Medicine, 6670 Bertner Ave, Houston, TX 77030, USA; ^2^Houston Methodist Hospital, Department of Quality, PI and Patient Safety, 6565 Fannin St, Houston, TX 77030, USA

## Abstract

**Background:**

The QuantiFERON Gold In-Tube (QFT-G) assay is used to identify individuals with tuberculosis infection and gives quantitative and qualitative results including positive, negative, or indeterminate results (that cannot be interpreted clinically). Several factors, including immunosuppression and preanalytical factors, have been suggested to be significantly associated with indeterminate QFT-G results. An online education program was designed and implemented to reduce the rate of indeterminate QFT-G test results at Houston Methodist Hospital (HMH).

**Methods:**

Data from patients' electronic medical records having indeterminate QFT-G results between 01/2015 and 05/2016 at HMH in Houston, TX, were administratively extracted for (1) medical unit where QFT-G phlebotomy was performed, (2) demographics, and (3) ICD-9/10 diagnosis codes. Unit nurses identified with high proportions of indeterminate QFT-G results were emailed a link to an online pretest educational program with a QFT-G blood collection and handling presentation, and a posttest assessment.

**Results:**

Of the 332 nurses emailed, 94 (28.4%) voluntarily completed both tests within the 6-month time allotted. The nurses that completed the education program had a significantly higher posteducation test score than on the pretest (70.2% versus 55.3%,* p*<0.001, effect size=0.82). Improved posttest score was seen in 67.0% of participants. No reduction in the proportion of indeterminate test results was seen overall at HMH in the 6 months after education.

**Conclusions:**

A targeted education program was able to successfully increase nurses' knowledge of blood collection and handling procedures for the QFT-G test, but no association was found between the improvement of posttest score and indeterminate QFT-G test results.

## 1. Background

Tuberculosis (TB) is the world's deadliest infectious disease, claiming the lives of three people every minute [1]. Identifying and treating persons with TB infection (TBI) are the prioritized strategies for TB control and prevention in the US [2]. Interferon gamma release assays (IGRAs) are indirect* in vitro* blood assays that use a cell-mediated immune response to test for TBI. The commercially available IGRAs include an ELISA-based test, the QuantiFERON-TB Gold In-Tube (QFT-G) assay (Qiagen Inc., Germantown, PA), an ELISpot test called the T-SPOT.*TB* (TSPOT) assay (Oxford Immunotec*¸* Abingdon, United Kingdom), and the newly FDA approved QuantiFERON Gold Plus assay (QFT-P).

QFT-G is a heparinized whole blood assay that utilizes overlapping polypeptides of* Mycobacterium tuberculosis* (*Mtb)* specific antigens to elicit interferon-gamma (IFN-*γ*) production from effector T cells that, in* Mtb *infected individuals, are capable of responding to these antigens. This three-tube assay is comprised of a positive control (phytohemagglutinin; PHA) tube, a negative control tube, and the antigen tube. The manufacturer “coats” the TB antigens and PHA onto the inside walls of the antigen and positive control tubes, respectively. Because of antigen “coating,” it is necessary to shake assay tubes to dissolve and mix the antigens with the blood to begin the reaction [3].

In a clinical setting, it is generally accepted that an indeterminate QFT-G result cannot be interpreted. Blood must be redrawn and the IGRA rerun which wastes resources and requires an additional blood draw. Studies have shown patient factors, including age and immunosuppression [4–10], and procedural errors such as blood collection by a nonphlebotomist [10, 11], variations in blood collection volume, thoroughness when shaking the tubes, and duration between collection and processing may potentially affect test results [12–14].

The study was conducted as a quality assurance project on all the QFT-G assays performed at Houston Methodist Hospital (HMH). After extensive analysis of indeterminate test results, it was hypothesized that preanalytical procedural lapses were leading to excess indeterminate QFT-G results from patients in HMH's inpatient medical units. Multiple meetings between performance improvement leadership, nursing managers, and laboratorians led to the creation of a modified education program [15–17] for nurses at HMH. Inpatient medical units with high proportions of indeterminate QFT-G test results were selected for a pilot education program for nurses in an attempt to reduce the indeterminate QFT-G test results. A survey and presentation focusing on QFT-G assay procedures including blood collection, tube shaking, and transportation to the laboratory were created to address potential preanalytical factors that may have contributed to the high proportion of indeterminate QFT-G test results. This pilot study was conducted to determine the effectiveness and utility of the education program at HMH in lowering the indeterminate rate of QFT-G's conducted among inpatient medical units.

## 2. Methods

### 2.1. Study Design and Sample

QFT-G tests with indeterminate results at HMH were identified from January 2015 to May 2016. Patients' medical records were mined retrospectively for the medical unit responsible for the phlebotomy, patient's age, gender, and primary/secondary ICD-9/ICD-10 codes. Patients' ICD-9/ICD-10 codes were queried for codes identifying the patients who were immunocompromised, as defined by the Agency for Healthcare Research and Quality, at the time of the QFT-G assay blood draw. The proportion of indeterminate QFT-G test results was calculated for each medical unit.

### 2.2. Measures

A survey was constructed using the REDCap online database (Vanderbilt University, Nashville TN). The survey included a pretest evaluation consisting of five questions concerning nurses' work experience and 11 multiple choice questions regarding the QFT-G, a PowerPoint presentation focusing on QFT-G blood collection, tube shaking, and transportation procedures, and a posttest evaluation consisting of the same 11 knowledge questions as the pretest presented in a different order, based on Qiagen's QFT-G training material [15–17]. The survey was private, so an individual survey link was sent to each invited participant's email. Participants could only take the survey once. After completing the pretest survey, participants were linked to the presentation and the postsurvey questionnaire. The educational program was assigned to nurses in the nine medical units at HMH with high proportions of indeterminate QFT-G test results in 2015 through May 2016. The survey invitations were emailed in September 2016, and participants were given approximately six months (9/26/2016–2/14/2017) to complete the survey. In-hospital QFT-G test results with phlebotomy from 2/09/2017 to 9/14/2017 were analyzed to determine if the education had reduced the proportion of indeterminate QFT-G test results in the hospital overall and in the participating medical units.

### 2.3. Statistical Analysis

Improvements in total test scores and in scores of individual questions between baseline (pretest) and post intervention (posttest) were compared using the paired t-test. The effect sizes indicating the magnitude of the differences were estimated using Cohen's* d* method [18]. The proportions of indeterminate QFT-G test results before and after the education program were compared for HMH overall and stratified by medical units that received or did not receive the education. All analyses were conducted using SAS 9.3 (SAS Institute Inc., Cary, NC). A p value <0.05 was considered statistically significant.

### 2.4. Ethics Statement

This study was conducted with the approval of the HMH's Quality Control Department. A waiver was obtained from the HMH's Institutional Review Board for this performance improvement project.

## 3. Results

### 3.1. Patient's with Indeterminate Test Results

Examination of QFT-G data obtained at HMH from January 2015 through May 2016 identified that 24.0% (175/728) of QFT-G tests were indeterminate within inpatient medical units where primary care nurses perform phlebotomy. By contrast, the indeterminate rate was 0.4% (11/2974) for QFT-G test results during the same time period when phlebotomy occurred by trained phlebotomists in the HMH outpatient laboratory. Proportions of indeterminate QFT-G test results in the medical units ranged from 0.6% to 10.3%. Due to the major causes of indeterminate QFT-G results being related to patient factors or preanalytical procedural errors, patient diagnostic codes were evaluated for immunocompetence. Approximately 37% of the patients with indeterminate results were immunocompromised based on ICD-9/10 primary and secondary codes, and there was no significant difference in the median IFN-*γ* measured in the positive and negative controls between immunocompromised and immunocompetent patients indicating that the majority of indeterminate test results were likely caused by preanalytical errors and not patient factors (Table 1). QFT-G were processed and analyzed at the onsite Molecular TB laboratory, so it was deemed unlikely that extreme environmental temperatures or freezing of blood samples were the cause of indeterminate QFT-G results.

### 3.2. Nursing Education Intervention

The education program was offered to nurses from nine inpatient medical units that accounted for 50.3% (88/175) of the original indeterminate QFT-G test results between January 2015 and May 2016. A total of 148 nurses of the 332 nurses employed in the selected units began the QFT-G education program. Of the 148 nurses who started the survey, 125 nurses completed the preeducation questionnaire (37.7%), and 94 nurses completed the posteducation questionnaire in the time allotted (28.3%). Of the 238 nurses that did not complete the survey, 16 ended employment at HMH during the program period, and one nurse refused participation in the survey due to never drawing blood for the QFT-G test. Rates of participation by nurses that completed the program by unit ranged from 18.5% to 52.9%. Of the 94 nurses that completed both the preeducation and the posteducation surveys, 58.5% (55/94) had been working as a nurse for more than five years, but 25.5% had been working in their current medical unit for less than one year (Table 2). Nurses commonly drew blood for laboratory tests, and 86.2% (81/94) reported drawing blood one or more times a week. However, 59.6% (56/94) reported drawing blood for the QFT-G approximately once in a six-month period (Table 2).

**Figure 1:**
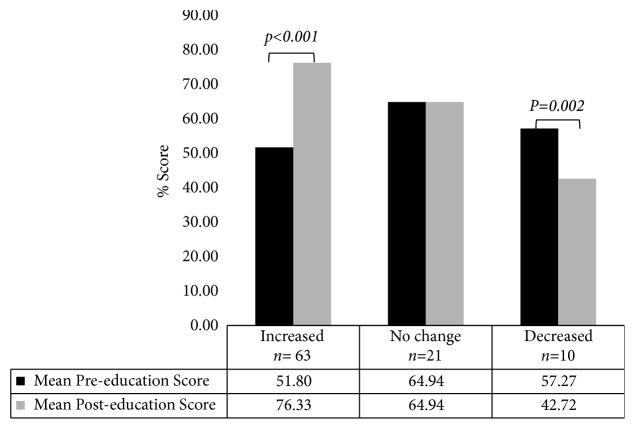
Mean preeducation and posteducation QFT-G knowledge test scores.

The overall mean score on the preeducation survey was 54.1% (median: 54.5%, IQR 45.5%, 63.6%) for nurses completing the preeducation questionnaire. For nurses completing the preeducation and the posteducation survey, the mean score of the preeducation questionnaire was 55.3% (median: 54.5%, IQR 45.5%, 63.6%), and the mean score of the posteducation questionnaire was 70.2% (median: 72.7%, IQR 54.5%, 81.8%) (Table 3). The education program led to a 14.9% increase in mean survey scores and a significant increase in knowledge due to the QFT-G education presentation (*p*<0.001, effect size = 0.82, Table 3). The majority of nurses (67.0%,* n*=63/94) had an improved score on the posteducation survey compared to the preeducation questionnaire, and the mean posteducation questionnaire score was significantly higher than the preeducation score (Figure 1).

The most common incorrectly answered question on the preeducation and posteducation questionnaire was “Is the IGRA test FDA approved for persons who have been identified with TB disease?” (74.4%, 93/125 and 73.4%, 69/94, respectively) (Table 4). The second most common incorrectly answered question on the preeducation survey was “What is the acceptable volume for QFT blood collection tubes?” (73.6%, 92/125; Table 4). Several questions concerning blood collection procedures saw significant increases in the score from the preeducation to posteducation questionnaires (Table 4).

### 3.3. Posteducation Analysis

There were 212 QFT-G tests administered within inpatient medical units during the posteducation period. Of those QFT-G tests, 64 (30.2%) had an indeterminate test result, and 28 (13.2%) had a positive test result. Indeterminate QFT-G test results occurred in 27 medical units during the 6-month period after education, and 32 (50.0%) indeterminate QFT-G test results were in medical units that received the education intervention. The two nursing units with the highest proportion of indeterminate test results in the analyzed preeducation period retained the same status 6 months after the education intervention was completed. These two inpatient medical units accounted for 31 of 175 (17.7%) indeterminate QFT-G test results in the preeducation period and 19 of the 64 (29.7%) indeterminate QFT-G test results in the posteducation period. These units had a 19.0% and 26.1% proportion of nurses complete the voluntary education program, and the proportion of indeterminate results remained over 40% of all QFT-G administered within the unit in the posteducation period. In addition, these units had a total of 44 (25.1%) QFT-G tests administered in the posteducation surveillance period of which 19 had indeterminate test results. Of the nine units given the education program, four showed a decrease in the proportion of indeterminate QFT-G test results, three showed no change in the proportion, and two had increases in the proportion of indeterminate QFT-G test results. The unit with the highest proportion of nurses that completed the education program (52.9%) had a decrease in the proportion of indeterminate QFT-G results from 5.7% in the preeducation analysis period to 1.6% in the posteducation analysis period.

## 4. Discussion

There was a significant overall improvement in the mean score between pre- and posteducation surveys (70.2% versus 55.3%,* p*<0.001, effect size = 0.82) in participants that completed the education program. The large effect size indicates the high effectiveness in implementing the education program to improve the nurse knowledge related to QFT-G testing despite the lack of a corresponding decrease in frequency of indeterminate assay results and poor response rate. Most participants (67.0%) completing the education program had a higher score for the posteducation questionnaire than the preeducation questionnaire indicating an increase in knowledge of QFT-G blood collection and handling procedures; however, 10 participants had a lower posteducation score than preeducation score. We believe nurses may have randomly selected answers to the survey and not that the education program confused the participating nurses. This study had a low response rate from invited nurses; less than 30% of nurses from nine selected in-patient medical units at HMH voluntarily completed the QFT-G education program in the allotted time (six months) when using an emailed invitation system for a voluntary education program. The 2 units with the highest proportion of indeterminate test results in the preeducation period both had low participation by nurses. Future education efforts may require the education program to be mandatory or attached to other education efforts, and alternate delivery methods need to be investigated.

The most common incorrectly answered question was a question regarding the purpose of the QFT-G assay. Most nurses believed that the assay was used for identification of TB disease when the test is FDA approved for the detection of* Mtb* infection. The next most common incorrectly answered question on the preeducation questionnaire (73.6%) involved blood collection procedures. Specifically, the question asked for the acceptable amount of blood collected in each assay tube. The most common answer was 1 mL and the correct answer was 0.8 mL to 1.2 mL as was validated in studies [12]. The QFT-G tubes have a thick black line on the label to indicate the 1 mL mark, and the tubes contain a vacuum to draw exactly 1 mL of blood. It is unlikely that they are trained to collect a variable volume of blood, so multiple-choice answer options for the question should be modified in the future to better reflect the nurses' training.

The increased information the nurses acquired on the QFT-G blood collection procedures due to the education intervention failed to translate to a reduction in the proportion in indeterminate test results. The overall proportion of indeterminate QFT-G in in-hospital medical units at HMH rose by 5.2% from preeducation period to the 6 months' posteducation period (24.0%-30.2%). The increased proportion may be due to the smaller number of QFT-G that were run in the shorter time observed in the posteducation period compared to the preeducation period (six months versus 17 months). There was no change in the proportion of indeterminate QFT-G among the nine medical units given the intervention after education, and the two units with the highest proportions of indeterminate test results retained that status in the posteducation period indicating the ineffectiveness of the targeted education intervention.

A large proportion of participating nurses (81.9%) indicated that they collected blood for the QFT-G test once every three to six months. This infrequency of drawing blood for the QFT-G test with its special instructions contributed to procedural lapses. Physicians most likely do not order the QFT-G test often enough for the assay to become familiar to the nurses. An alternative to continuous education may be to dedicate a phlebotomist to draw blood for the QFT-G as is done in the outpatient center that has a low indeterminate rate. At HMH, the outpatient phlebotomy lab draws blood for the QFT-G at a greater frequency than the inpatient units. There are fewer phlebotomists drawing blood for a greater number of QFT-G assays compared to the large number of nurses in the inpatient units drawing blood at a lower frequency for the assay. Reenforcing training through refresher courses may be able to reenforce nurses' knowledge of the QFT-G assay.

At HMH, effort has been made to ensure that proper QFT-G procedures have been followed. An instruction sheet is prepacked with the assay tubes detailing times of collection, amount of blood to collect, and temperature to store and transport tubes to the laboratory. The laboratory technicians inspect all QFT-G assay tubes blood volume and blood collection time to ensure incubation occurs within 12 hours of phlebotomy. Education on the QFT-G blood collection and handling is included in the new hire nurses' training; however, in no other routine education efforts is this training/education repeated. Nurses are also routinely educated to be gentle with drawn blood and instructed to gently invert tubes by rotating their wrists to mix blood [19, 20] to avoid vigorous shaking thus preventing hemolysis. This mixing method is inadequate for the QFT-G, which requires that blood fully coat the inside surface of the tube to fully mix the antigens within the specimen tube [3].

Our results were supported by the literature. A study conducted at a local children's hospital found that 31% (56/182) of QFT-G assays had an indeterminate result, and the authors concluded that improper specimen handling was the likely cause [11]. A study at the Cleveland Clinic to analyze the potential reasons for an excess of indeterminate QFT-G test results (11% versus the expected 5%) found that preanalytical factors of prolonged incubation, overfilling of tubes, and inadequately shaken tubes were primarily responsible for the excess indeterminate test results [21].

A strength of this study was that the education material was designed to address preanalytical procedural lapses identified specially at HMH. The study was supported by the Performance Improvement/Quality Control Department and medical unit directors. Limitations of the study included the low response rate to the emailed invitations for voluntary education despite the support from unit directors. Incentives and in-person reminders to the medical units resulted in an increase in participation, but the overall response rate remained low. Little to no data was available on the clinical diagnosis and reasons for the QFT-G request from outpatient visitors to HMH. The lack of patient data on the QFT-G assay conducted during the preeducation analysis period in the outpatient clinic prevented the authors from pursuing an in-depth analysis on patient factors that may be associated with indeterminate QFT-G results. The authors were also prevented from conducting a comparison of factors that differentiate the inpatient and outpatient groups with indeterminate QFT-G results because of this lack of data. A relatively small number of QFT-G tests were administered in the posteducation surveillance period which can lead to skewed results due to a few outliers. The study lacked data on reason for ordering the QFT-G assay beyond the clinician “ruling-out” TB infection or disease as part of the diagnostic differential. Due to the small sample size and limited data on demographics, clinical diagnosis, and reason for ordering the QFT-G assay, a stratified analysis by medical unit was not able to produce meaningful results.

Due to the low participation rate and the inability of the education program to decrease the rate of indeterminate QFT-G among inpatient medical units, the pilot education intervention has not been continued at HMH. Having to repeat the QFT-G on nearly a quarter of patients tested increases costs, wastes resources, inconveniences patients, and potentially delays treatment. A new option is the QFT-P test that has recently received FDA approval in the US. For the QFT-P, blood can be drawn directly into the assay tubes as with the QFT-G or phlebotomized into heparinized tubes [22]. After delivery to the laboratory, blood is transferred from a lithium heparin tube to the QFT-P assay tubes and shaken by laboratory technicians to start the assay reaction. This process would remove the need to train nurses to vigorously shake the QFT-G tubes after blood collection.

## 5. Conclusion

We recommend that primary hospitals such as HMH enact a surveillance system to monitor the proportion of positive, indeterminate, and negative QuantiFERON assays. Processes should be monitored to ensure that samples are collected, handled, stored, and transported according to manufacturer's guidelines. Regular education and training of nursing staff or phlebotomists that collect blood for the QuantiFERON assay on the correct blood collection and handling procedures for the QuantiFERON test may aid in reducing the rate of indeterminate results due to improper preanalytical procedures. Having dedicated phlebotomists assigned to draw blood for the QuantiFERON assay could also reduce the number of staff that need to be trained. Programs may decide if the cost of dedicated phlebotomists may be balanced by cost savings of reduced indeterminate QuantiFERON assays. Hospitals and other clinics now have the option of converting to phlebotomy collected into a single lithium heparin tube that is transferred to QFT-P assay tubes by laboratory staff, which would increase their workload.

## Figures and Tables

**Table 1:** Characteristics of patients with indeterminate QFT-G results before education program was implemented.

	**N=175**		
**Patients Characteristics**	***n***	%	

Gender			
Female	86	49.1%	
Male	89	50.9%	
Immunocompromised			
Yes	65	37.1%	
No	110	62.9%	
Age, median (IQR)	55	(42, 66)	

**Indeterminate Characteristics**	Immunocompetent	Immunocompromised	*P* value
Nil value, median (IQR)	0.03 (0.02, 0.05)	0.04 (0.02, 0.08)	0.229
Mitogen value, median (IQR)	0.15 (0.07, 0.31)	0.14 (0.06, 0.29)	0.424

**Table 2:** Characteristics of nurses that completed the QFT-G education program.

**Characteristic**	**N=94**	%
**Years Nursing**		
Less than 1	4	4.3
1-2	18	19.2
3-5	17	18.1
≥5	55	58.5
**Years Worked in Current Unit**		
Less than 1	24	25.5
1-2	33	35.1
≥3	37	39.4
**Frequency draw blood for a laboratory test**		
≥ 1 per Week	81	86.2
≥ 1 per Month	6	6.4
1 per 3 Month	5	5.3
1 per 6 Month	2	2.1
**Frequency draw blood for a QuantiFERON test**		
≥ 1 per Week	3	3.2
≥ 1 per Month	14	14.9
1 per 3 Months	21	22.3
1 per 6 Months	56	59.6

**Table 3:** Changes in mean score between pre- and posteducation questionnaire.

**Overall**	***n***	**Mean Score**	**SD**	***P *Value**	**Effect size**
Pre-education	94	55.3%	17.16%	<0.001	0.82
Post-education	94	70.2%	18.96%		

Analysis was conducted on nurses that completed pre- and posteducation questionnaire; SD, standard deviation; effect size was estimated using Cohen's d method.in analysis.

**Table 4:** Frequency of incorrectly answered questions and mean score and effect size of questions on preeducation and posteducation questionnaire.

	**Number of Nurses Answered Incorrectly** ***n* (**%**)**		**Mean total score (±SD)**			
**Questions**	**Pre-education** **(*n*=125)**	**Post-education** **(*n*=94)**	**Pre-education**	**Post-education**	***P* Value**	**Effect size**
What is the QuantiFERON Gold test used for?	14 (11.2)	3 (3.19)	89.4 (±31.0)	96.8 (±17.7)	0.034	0.30
What is the acceptable volume for QFT blood collection tubes?	92 (73.6)	42 (44.7)	29.8 (±46.0)	55.3 (±50.0)	<0.001	0.53
After blood is collected into the QFT blood collection tubes, what is the next step in the process?	9 (7.2)	15 (16.0)	92.6 (±26.4)	84.0 (±36.8)	0.059	0.27
What is the purpose of shaking the QFT blood collection tubes?	82 (65.6)	49 (52.1)	35.1 (±48.0)	47.9 (±50.2)	0.018	0.26
At what temperature do you store the tubes until they are delivered to the laboratory?	80 (64.0)	30 (31.9)	36.2 (±48.3)	68.1 (±46.9)	<0.001	0.67
What is the proper order for filling the blood collection tubes?	75 (60.0)	19 (20.2)	41.5 (±49.5)	79.8 (±40.4)	<0.001	0.85
Is frothing of blood in the QFT blood collection tubes normal after the shaking process?	29 (23.2)	15 (16.0)	79.8 (±40.4)	84.0 (±36.8)	0.374	0.11
Choose the appropriate phrase to complete the following sentence: When a “butterfly needle” is being used to collect blood, which is often done when many tubes require blood collection....	69 (55.2)	35 (37.2)	46.8 (±50.2)	62.8 (±48.6)	0.008	0.32
Can blood be drawn from a PICC or Central line for the QFT?	57 (45.6)	16 (70.0)	55.3 (±50.0)	83.0 (±37.8)	<0.001	0.62
Is inverting the tubes once an acceptable mixing method immediately after blood collection?	31 (24.8)	15 (15.96)	77.7 (±41.9)	84.0 (±36.8)	0.181	0.16
Is the IGRA test FDA approved for person who has been identified with TB disease?	93 (74.4)	69 (73.4)	24.5 (±43.2)	26.6 (±44.4)	0.640	0.05

Pre- and posttest comparison calculated by paired t-test in nurses who completed both pre- and posttests (N=94). Effect size was estimated using Cohen's d method; QFT, QuantiFERON Gold In-Tube; PICC, peripherally inserted central catheter; IGRA, interferon-*γ* release assay; FDA, US Food and Drug Administration; TB, tuberculosis.

## Data Availability

The patient data and pre- and posttest results used to support the findings of this study are restricted by the Houston Methodist Hospital Institutional Review Board in order to protect patient and employee privacy.
